# Vascular plants dataset of the herbarium (COFC) of the University of Cordoba, Spain

**DOI:** 10.3897/phytokeys.133.37481

**Published:** 2019-10-15

**Authors:** Gloria Martínez-Sagarra, Juan Antonio Devesa

**Affiliations:** 1 Department of Botany, Ecology and Plant Physiology, University of Cordoba, Rabanales Campus, 14071, Cordoba, Spain University of Cordoba Cordoba Spain

**Keywords:** Western Andalusia, Cordoba, COFC, herbarium collection, "Flora iberica", Spain, taxonomy, University of Cordoba, vascular plants

## Abstract

This paper describes the herbarium (COFC) dataset of vascular plants of the University of Cordoba (SW Spain). This dataset is made up of two collections, the General collection (61,377 specimens) and the Historical collection (1,614 specimens). This study has focused mainly on the General collection, which contains the largest number of vascular plant specimens, predominantly angiosperms, mainly provincial and regional (Andalusia, Spain), but also with a good representation of other areas of the Iberian Peninsula and neighboring countries. The place of collection is specified in 99.7% of the labels, about 35% being georeferenced, and it is estimated that, currently, about 86% of the material housed in the herbarium has been databased using Elysia v1.0. software. With more than 178 families, 1,178 genera, and 3,750 species, this collection not only has educational importance, but is a valuable research tool that has been useful for the development of important works such as "Flora Vascular de Andalucía Occidental" and the "Flora iberica". The dataset described in this paper is registered with GBIF (accessible at https://doi.org/10.15468/fdzzal).

## Context of the COFC herbarium

The herbarium (COFC) of the University of Cordoba (Spain) is located in the Rabanales Campus, on the outskirts of the city of Cordoba. It was created in 1977 and is associated with the Department of Botany, Ecology and Plant Physiology (Botany Section, Faculty of Sciences). The herbarium, of which Prof. J.A. Devesa is currently the curator, is registered in the Index Herbariorum with the acronym COFC (Thiers 2019, http://sweetgum.nybg.org/science/ih/). It includes three botanical collections: one of fungi (Fungi-COFC, 4,827 specimens) and two of vascular plants, the Historical collection (1,614 specimens) and the General collection (61,377 specimens), the latter being the most significant. To these will be added in the future the recently created collections of bryophytes and algae, in the initial phase and barely significant. So far, 98.7% of the specimens have been databased and they can be accessed through the GBIF network (http://data.gbif.org). Some interesting data from the Historical collection are provided, but the dataset described in this paper covers exclusively the General collection.

### Historical collection

The Historical collection, created by the multifaceted man of religion José de Jesús Muñoz Capilla (who lived 1771−1840), contains a total of 1,614 specimens of vascular plants arranged in 22 classes according to the Linnaean system (Infante et al. 2002). Most of the specimens were collected during the years 1792−1834, mainly in the province of Cordoba, including some from plants cultivated in gardens, but occasionally in other provinces (e.g., Seville, Jaen, Cadiz, and Madrid), by the following botanists from Cordoba: Jesús Muñoz Capilla, Rafael Mariano León y Gálvez (1772−1811), Rafael Entrena y Camacho (1786−1835), and the Cádiz-born Antonio Nicolás Cabrera y Corro (1762−1827). This collection was ceded by the relatives of Muñoz Capilla in the mid-nineteenth century to the Special School of Veterinary Medicine (González Soriano 1923), an institution that more recently would become the Veterinary Faculty and the germ of the current University of Cordoba. The herbarium was restored and carefully inventoried by Jordano and Ocaña (1955, 1957), and in the 1970s, after the creation of the University of Cordoba, it was ceded to the facilities of the COFC herbarium. The collection houses some 900 species; only 17% of the labels provide some information about the place of collection, which in many cases is limited to the province, and for only about 5% is the year of collection stated. Its importance lies in its historical and cultural value, since it also contains plants collected by and/or annotations of important botanists with whom it maintained contact (Laza Palacios 1944), among them Mariano Lagasca (1776−1839), Félix Haenseler (1767−1841) and Edmond Boissier (1810−1885). The data in this collection can be consulted at https://doi.org/10.15468/f3tcb6.

### General collection

The General collection has a total of 61,377 specimens that have been recorded with different herbarium number, and there are more than 12,000 duplicate specimens of the gatherings. This collection is the fourth largest among the Andalusian collections in terms of the number of specimens (based on the information obtained from the National Biodiversity Information Node 2015), after SEV, COA and MGC (Table 1). It contains material collected for the most part over the last 40 years from explorations and studies carried out by researchers from the Department of Botany, Ecology and Plant Physiology, undergraduate and post-graduate studies related to teaching and, in minor measure, exchanges with or donations by other herbaria. The collection comprises a wide representation of vascular plants – especially angiosperms− from the province of Cordoba and, to a lesser extent, from other Spanish regions, as well as a modest representation from bordering countries and other parts of the world. It mainly includes wild plants, with a minimum representation of ornamental plants (around 950 specimens). In recent years the collection has been enhanced, mainly by a large number of collections made in the Iberian Peninsula for taxonomic revision of various genera, especially *Centaurea* L. (Asteraceae) and *Festuca* L. (Poaceae), within the "Flora iberica" project. This is one of the Spanish herbaria with greater representation of these genera. The herbarium, therefore, includes an important sample of the vascular plant biodiversity of the Iberian Peninsula, especially that of Western Andalusia (SW Spain), so it is not only essential for the knowledge and study of local flora, but also has been very useful for "Flora de Andalucía Occidental" (Valdés et al. 1987) and, currently, the "Flora iberica" (Castroviejo 1986−2019, http://www.floraiberica.org/).

**Table 1.** The main Andalusian herbaria (Source: https://www.gbif.org, accessed 2019). (*) Information from the National Biodiversity Information Node (2015), available at https://www.gbif.es/wp-content/uploads/2018/02/GBIF.ES_Inf_Col_2014.pdf. Acronyms of the herbaria according to Thiers (2019).

Acronyms	Collection	Institution	Estimated number of specimens (year of estimate)*	Number of records published in GBIF (accessed 2019)
SEV	Herbarium of the University of Seville	University of Seville	350,000 (2007)	226,498
COA	COA Herbarium	Botanical Garden of Cordoba	80,000 (2008)	39,835
MGC	MGC-Cormof Herbarium	Department of Plant Biology, Faculty of Sciences	74,683 (2012)	78,930
COFC	COFC Herbarium of the University of Cordoba: General collection	Department of Botany, Ecology and Plant Physiology, Faculty of Sciences	72,000^1^ (2019)	60,566
GDA	Collection of vascular plants of the herbarium of the University of Granada	University of Granada	1,442 (2011)	55,219^2^
HUAL	Herbarium of the University of Almeria	University of Almeria. Department of Plant Biology and Ecology	24,304 (2012)	14,971
JAEN	Herbarium of vascular plants	University of Jaen. Faculty of Experimental Sciences	34,000 (2012)	No data
UPOS	Herbarium of the University Pablo de Olavide	University of Pablo de Olavide	10,000 (2008)	No data

^1^ Estimation of sheets of unmounted and unfiled specimens; ^2^ It including the GDAC herbarium.

This collection regularly increases, by about 1,250 specimens per year, and the dataset is periodically uploaded to the GBIF portals. The data referring to this collection can be found at https://doi.org/10.15468/fdzzal (entries through 2018−12−31), except for the data related to the genus *Festuca*, which have not been uploaded yet since they are still under study.

## Taxonomic coverage

The collection of vascular plants has 61,377 specimens belonging to 178 families, 1,178 genera, and 3,750 species. Of the specimens in the collection, 97.8% are identified at the species level and the remaining 2.2% (about 1,350 specimens) only at the level of the genus, awaiting their review by a specialist.

**Figure 1.**
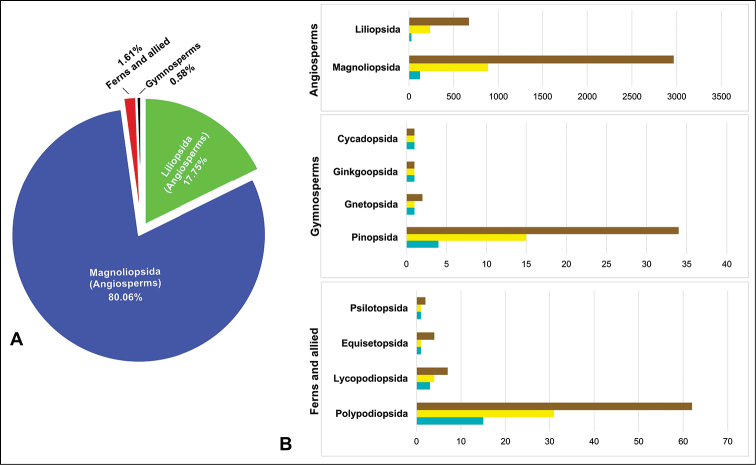
Taxonomic coverage of the General collection dataset in terms of specimens (**A**). Number of species (brown), genera (yellow), and families (light blue) within each class (**B**).

The majority of the specimens in the collection are angiosperms (Angiospermae) belonging to the classes Magnoliopsida (49,140 specimens; 80.06%) and Liliopsida (10,893 specimens; 17.75%). These are followed, in terms of representation, by ferns and related groups, with 991 specimens (1.61%) and the following distribution: class Polypodiopsida (740 specimens), Equisetopsida (139 specimens), Lycopodiopsida (107 specimens), and Psilotopsida (5 specimens). Finally, for gymnosperms (Gymnospermae) there are 353 specimens (0.58%), with the following distribution: Cycadopsida (2 specimens), Ginkgoopsida (1 specimen), Gnetopsida (51 specimens), and Pinopsida (299 specimens). In terms of diversity, Magnoliopsida (124 families, 886 genera, and 2,966 species), Pinopsida (4 families, 15 genera, and 34 species), and Polypodiopsida (15 families, 31 genera, and 62 species) are the most diverse classes within each large group of vascular plants (Fig. 1).

**Figure 2.**
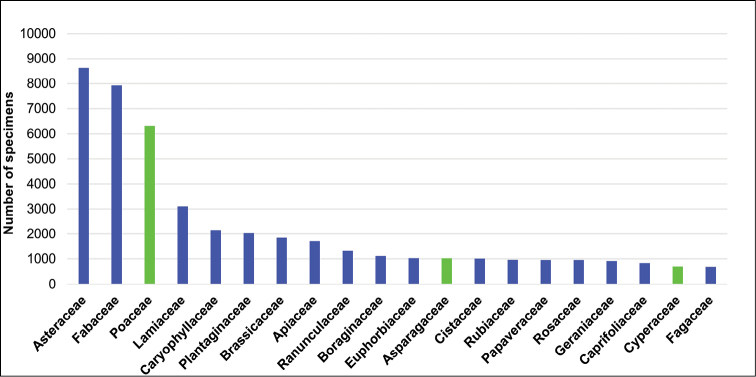
Families with greater representation in the General collection. In blue, families of Magnoliopsida; in green, families of Liliopsida.

**Figure 3.**
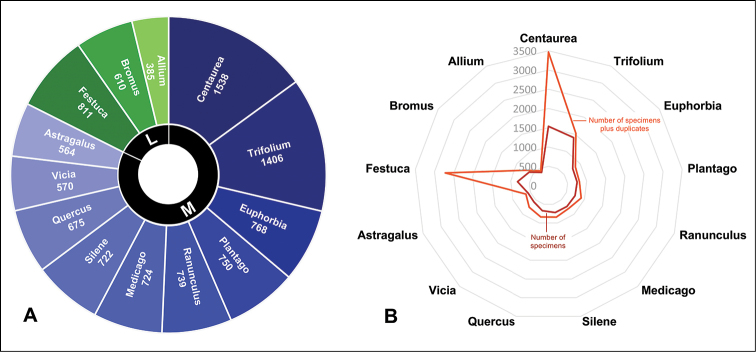
Genera with greatest number of specimens in the General collection (**A**). In blue, genera of Magnoliopsida (M); in green, genera of Liliopsida (L). Number of specimens collected for each genus plus associated duplicates (**B**).

The ten families that contribute most, in percentage terms, to the plant specimens conserved in the herbarium are: Asteraceae (8,625 specimens; 14.05%), Fabaceae (7,929 specimens; 12.92%), Poaceae (6,324 specimens; 10.30%), Lamiaceae (3,105 specimens; 5.06%), Caryophyllaceae (2,156 specimens; 3.51%), Plantaginaceae (2,033 specimens; 3.31%), Brassicaceae (1,851 specimens; 3.02%), Apiaceae (1,707 specimens; 2.78%), Ranunculaceae (1,319 specimens; 2.15%), and Boraginaceae (1,111 specimens; 1.81%) (Fig. 2). Among the best represented genera are *Centaurea* (1,538 specimens), *Trifolium* (1,406 specimens), *Festuca* (811 specimens), *Euphorbia* (768 specimens), *Plantago* (750 specimens), *Ranunculus* (739 specimens), *Medicago* (724 specimens), *Silene* (722 specimens), *Quercus* (675 specimens), and *Bromus* (610 specimens) (Fig. 3). The two genera with the highest specific diversity are *Centaurea* (102 spp.) and *Festuca* (59 spp.), followed by *Trifolium* (41 spp.), *Silene* (40 spp.), *Euphorbia* (37 spp.), *Ononis* (37 spp.), *Genista* (35 spp.), *Ranunculus* (34 spp.), *Astragalus* (28 spp.), and *Teucrium* (27 spp.).

## Taxonomic ranks

**Kingdom**: Plantae

**Phylum**: Tracheophyta

**Class**: Cycadopsida, Equisetopsida, Ginkgoopsida, Gnetopsida, Liliopsida, Lycopodiopsida, Magnoliopsida, Pinopsida, Polypodiopsida, Psilotopsida.

**Family**: Acanthaceae, Adoxaceae, Aizoaceae, Alismataceae, Amaranthaceae, Amaryllidaceae, Anacardiaceae, Annonaceae, Apiaceae, Apocynaceae, Aquifoliaceae, Araceae, Araliaceae, Araucariaceae, Arecaceae, Aristolochiaceae, Asparagaceae, Asphodelaceae, Aspleniaceae, Asteraceae, Athyriaceae, Balsaminaceae, Begoniaceae, Berberidaceae, Betulaceae, Bignoniaceae, Blechnaceae, Boraginaceae, Brassicaceae, Bromeliaceae, Butomaceae, Buxaceae, Cactaceae, Campanulaceae, Cannabaceae, Capparaceae, Caprifoliaceae, Caryophyllaceae, Casuarinaceae, Celastraceae, Ceratophyllaceae, Cistaceae, Cleomaceae, Clusiaceae, Colchicaceae, Commelinaceae, Convolvulaceae, Coriariaceae, Cornaceae, Crassulaceae, Cucurbitaceae, Culcitaceae, Cupressaceae, Cycadaceae, Cymodoceaceae, Cynomoriaceae, Cyperaceae, Cystopteridaceae, Cytinaceae, Davalliaceae, Dennstaedtiaceae, Didiereaceae, Dioscoreaceae, Drosophyllaceae, Dryopteridaceae, Ebenaceae, Elaeagnaceae, Elatinaceae, Ephedraceae, Equisetaceae, Ericaceae, Escalloniaceae, Euphorbiaceae, Fabaceae, Fagaceae, Frankeniaceae, Garryaceae, Gentianaceae, Geraniaceae, Ginkgoaceae, Grossulariaceae, Haloragaceae, Heliotropiaceae, Hydrangeaceae, Hydrocharitaceae, Hypericaceae, Iridaceae, Isoetaceae, Juglandaceae, Juncaceae, Juncaginaceae, Lamiaceae, Lauraceae, Lentibulariaceae, Liliaceae, Linaceae, Linderniaceae, Lycopodiaceae, Lythraceae, Magnoliaceae, Malvaceae, Marantaceae, Marsileaceae, Martyniaceae, Melanthiaceae, Meliaceae, Menispermaceae, Menyanthaceae, Molluginaceae, Montiaceae, Moraceae, Musaceae, Myricaceae, Myrtaceae, Nephrolepidaceae, Nyctaginaceae, Nymphaeaceae, Oleaceae, Onagraceae, Ophioglossaceae, Orchidaceae, Orobanchaceae, Osmundaceae, Oxalidaceae, Paeoniaceae, Papaveraceae, Passifloraceae, Paulowniaceae, Phrymaceae, Phyllanthaceae, Phytolaccaceae, Pinaceae, Piperaceae, Pittosporaceae, Plantaginaceae, Platanaceae, Plumbaginaceae, Poaceae, Polemoniaceae, Polygalaceae, Polygonaceae, Polypodiaceae, Portulacaceae, Potamogetonaceae, Primulaceae, Proteaceae, Pteridaceae, Rafflesiaceae, Ranunculaceae, Resedaceae, Rhamnaceae, Rosaceae, Rubiaceae, Ruppiaceae, Rutaceae, Salicaceae, Salviniaceae, Santalaceae, Sapindaceae, Saxifragaceae, Scrophulariaceae, Selaginellaceae, Simaroubaceae, Smilacaceae, Solanaceae, Tamaricaceae, Taxaceae, Tetradiclidaceae, Thelypteridaceae, Thymelaeaceae, Tropaeolaceae, Typhaceae, Ulmaceae, Urticaceae, Verbenaceae, Violaceae, Vitaceae, Zygophyllaceae.

## Geographic coverage

**Figure 4.**
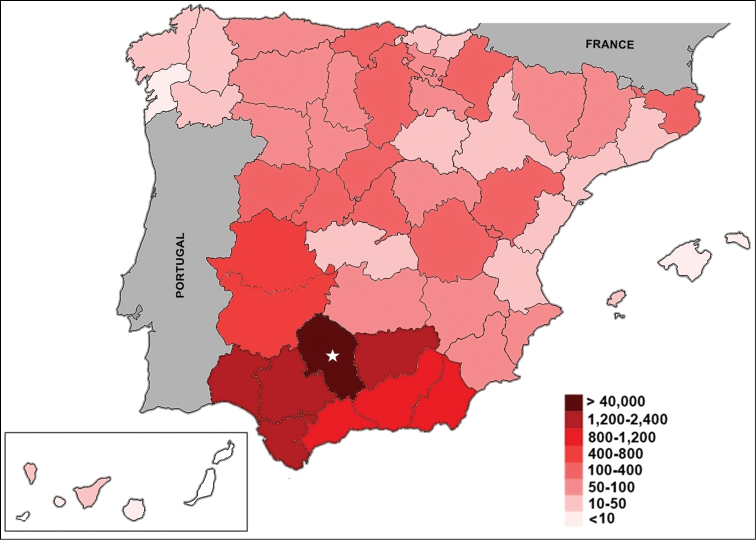
Geographical distribution (provincial representation) of specimens of the General collection in Spain. The star marks Cordoba province.

Most of the specimens in the collection are from Spanish territory (60,847 specimens; 99.14%), with the peninsular area being the best represented (Fig. 4). Less than 1% of the specimens come from bordering countries, such as Portugal (316 specimens), Morocco (185 specimens), and France (11 specimens). The specimens collected elsewhere −in Andorra (5 specimens), Norway (4 specimens), Switzerland (2 specimens), Great Britain (2 specimens), Ireland (1 specimen), Israel (1 specimen), Italy (1 specimen), and the USA (1 specimen) −, have a symbolic presence, representing only 0.03% of the collection.

Within Spain, Andalusia (Southern Spain) is the region with the highest number of specimens; specifically, within Andalusia, the province of Cordoba has the greatest representation (46,505 specimens), followed by Seville (2,244 specimens), Jaen (1,714 specimens), Huelva (1,640 specimens), and Cadiz (1,396 specimens) (Fig. 4).

**Figure 5.**
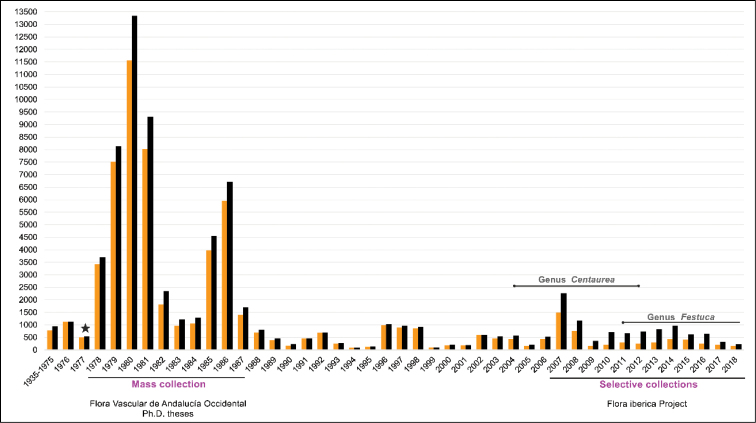
Number of specimens (orange), and specimens plus associated duplicates (black) collected between the years 1935 and 2018. The principal highlights are indicated, and the star marks the year of creation of COFC.

In the province of Cordoba, an important collection effort was made immediately after creation of the herbarium (Fig. 5). This has included all the territories of the Sierra Morena natural areas (northern half of the province), and the Campiña and Sierra Subbetica in the southern half. These include the territories of the Cardeña−Montoro and Sierra de Hornachuelos Natural Parks, in the Sierra Morena, and the Natural Park of the Sierra Subbetica and the Natural Reserves of the Humid Areas, in the South of Cordoba province.

## Temporal coverage

The collection includes specimens collected from 1935 to the present (Fig. 5). The oldest specimens correspond mainly to particular donations and exchanges, mainly involving renowned Spanish botanists such as José Cuatrecasas (1903−1996), Salvador Rivas Goday (1905−1981), Francisco Bellot (1911−1983), Bartolomé Casaseca (1920−1998), and José Vicente Cordeiro Malato-Beliz (1920−1993). The largest number of specimens (about 44,300 specimens, with 6,300 duplicates) was collected at the end of the 1970s and in the mid-1980s, coinciding with the years of creation of the University of Cordoba and COFC (Fig. 5). In this period, the source of the material was mainly PhD theses on flora and chorology, involving lecturers and contracted staff of the Department of Botany, Ecology and Plant Physiology, and also the surveys and taxonomic revisions of genera within the framework of the project "Flora Vascular de Andalucía Occidental" (Valdés et al. 1987). Subsequently, the material incorporated into the herbarium had its origin in more or less periodic collections carried out throughout the province of Cordoba, from which numerous chorological publications of local interest were derived. A new boost to the collection took place during the period 2007−2018 (about 4,900 specimens, with 4,600 duplicates, Fig. 5). In this case mainly due to the collections made in the Iberian Peninsula as part of the "Flora iberica" project (National Plan of R+D+i of the Government of Spain; see the later section Funding of the maintenance and databasing of the herbarium), and this is around 8% of the specimens that the collection houses.

On the other hand, as expected, high seasonality can be seen in the collections: more contributions are made in spring and summer than in winter. Thus, the months of greatest collection effort are April, May and June (almost 40,000 specimens); while the minor ones are December, January and November (only about 2,500).

## Step description

### Plant processing procedures

The usual procedures for the processing and storage in the herbarium of the plant material have been employed. The fresh material is dried by pressing, sometimes with simultaneous drying using an oven or heater. When it is dry, and prior to storage, the rotary freezing technique (at -18 °C) is used for four days in order to conserve the material and prevent its destruction by fungi and insects, thus avoiding subsequent infestations. This procedure is repeated approximately every six months. The specimens are stored in boxes inside compactor cabinets located in an isolation unit with controlled temperature and humidity. The families are arranged alphabetically, as are the genera within each family and the species within each genus.

### Labeling

Once processed, the specimens go along the assembly line, where they are assembled and labeled. Subsequently, they are assigned a COFC accession number and databased using Elysia v1.0. software (Pando et al. 1996−2016). It is estimated that 99.7% of the labels in the collection specify the place of collection. The information on the labels is heterogeneous as regards the origin of the material, and depends largely on the collector and his/her purpose. A complete label includes taxonomic information related to the identity of the sample (scientific name and authorship) and information on the place of collection: country, province, municipality, a more precise geographic location, GPS coordinates (and the projection system), altitude, ecological or descriptive data, date, field number, and collector(s) and identifier name(s).

### Quality control

The care and control of the collection includes its monitoring through the management and registration of all incoming and outgoing botanical gatherings that are the responsibility of the Herbarium Service, with the curator’s supervision. The process includes space planning, sample relocation, sample assembly and repair, freezing to minimize the potential for insect infestation, and general repairs. Periodic checks are made in the storage area of the collections to detect possible effects of insects and harmful fungi.

The quality control is mainly performed at three levels:

1. **In the identification phase/taxonomic criteria**: For their inclusion in the herbarium, the specimens must be identified at the level of the species and, if appropriate, the infraspecific category, although for many genera there is material without identification of the species that is waiting to be reviewed by specialists. The main works used to identify the material are: "Flora Vascular de Andalucía Occidental" (Valdés et al. 1987), "Flora Vascular de Andalucía Oriental" (Blanca et al. 2009), "Flora iberica" (Castroviejo 1986−2019), and "Flora Europaea" (Tutin et al. 1964−1980). In cases where a modification of the original identification is made, it is reflected in the database as a revision. Currently, work is being done on the organization and delimitation of families according to The Angiosperm Phylogeny Group III (APG III 2009) and IV (APG IV 2016). Regarding nomenclature, it is checked with several taxonomic authorities, such as The Plant List (http://www.theplantlist.org/), Tropicos (http://www.tropicos.org), IPNI (The International Plant Names Index, http://www.ipni.org/), and Euro+Med PlantBase (the information resource for Euro-Mediterranean plant diversity, http://ww2.bgbm.org/EuroPlusMed/), as well as the valid name recognized in "Flora iberica" (http://www.floraiberica.es/).

2. **Georeferencing and technical validation**: It is estimated that only 33% of the specimens in the collection contain information regarding the coordinates (UTM, MGRS, or geographic) of the place of origin, with greater or lesser precision. At present, the georeferencing of the collection is being done using MGRS coordinates (1 km^2^ of precision) based on the information contained in the original label, using the tools of Google Earth (http://www.google.com/earth/index.html) and Iberpix (http://www.ign.es/iberpix2/visor/). These coordinates are automatically transformed into geographic coordinates (latitude and longitude are expressed in decimal degrees in the WGS84 datum) when the herbarium information is transferred to the GBIF portal.

3. **In the phase of databasing and data export to the GBIF/Data records network**: The first stage of the databasing of the collection was carried out with Herbar v3.7.1 software (Pando et al. 1994−2010), but afterwards all the information in the collection was transferred to Elysia v1.0 software (Pando et al. 1996−2016), which is the one used today. It is estimated that around 86% of the material housed in the herbarium has been databased. The first transfer of data from COFC to the GBIF portal included data for the period 2006−2012 (56,081 records), and very recently the corresponding data up to and including 2018 were exported (a total of 60,566 records, excluding *Festuca*). The Darwin Core biodiversity standards were used (DarwinTest v3.3 tool; Ortega-Maqueda and Pando 2008) before uploading the information to the GBIF portal. The Darwin Core file (http://rs.tdwg.org/dwc/), encoded in UTF-8, is generated regularly and is available for download on the GBIF data portal via the Integrated Publishing Toolkit (IPT) of the University of Cordoba (version 1.8, published 2019; https://ipt.gbif.es/resource?r=herbariocofc#versions), through the GBIF Spanish network. The list of elements in the Darwin Core standard published through the GBIF network as well as their definitions and other information of interest can be found at https://dwc.tdwg.org.

## Funding of the maintenance and databasing of the herbarium

COFC herbarium does not yet have its own funding. Since its creation, it has been dependent on the personnel and resources of the Department of Botany, Ecology and Plant Physiology of the University of Cordoba. Only more recently has it received additional funding, fruit of the efforts made by the two Curators who have been in charge, Profs. E. Ruiz de Clavijo (1977−2010) and J. A. Devesa (2011−present). It had two grants at the beginning of the databasing of the collection (Complementary Funding of the Ministry of Education and Science), and since 2011 it has benefitted from the work of technical personnel hired for the maintenance, databasing, and georeferencing of the collection, in some cases financed by the Government of Spain, and in others by the Junta de Andalucía. These grants and contracts are summarized below.

### Funding received for the initial digitalization of the collection

**Funding body**: Ministry of Education and Science

**Reference**: CGL2007-28813-E

**Lead researcher**: E. Ruiz de Clavijo

**Duration**: January 2008 to December 2008

**Amount**: 32,900 euros

**Funding body**: Ministry of Education and Science

**Reference**: CGL2008-02241-E/BOS

**Lead researcher**: E. Ruiz de Clavijo

**Duration**: January 2009 to December 2009

**Amount**: 23,000 euros

### Contracts of the complementary actions framework

**Program**: Adaptation of the Herbaria of the Faculty of Sciences of the University of Cordoba to the GBIF framework (Subprogram: Complementary Actions)

**Funding body**: Ministry of Science, Innovation, and Universities

**Lead researcher**: E. Ruiz de Clavijo

**Duration**: July 2006 to April 2007

**Program**: Adaptation of the Herbaria of the Faculty of Sciences of the University of Cordoba to the GBIF framework (Subprogram: Complementary Actions)

**Funding body**: Ministry of Science, Innovation, and Universities

**Lead researcher**: E. Ruiz de Clavijo

**Duration**: January 2008 to December 2008

**Program**: Adaptation of the Herbaria of the Faculty of Sciences of the University of Cordoba to the GBIF framework (Subprogram: Complementary Actions)

**Funding body**: Ministry of Science, Innovation, and Universities

**Lead researcher**: E. Ruiz de Clavijo

**Duration**: March 2009 to December 2009 (extended until 30−10−2010)

Contracts for technical staff member:

**Program**: Technical Personnel to Assist Research

**Funding body**: Ministry of Science and Innovation

**Reference**: PTA2010-3438-I

**Lead researcher**: J. A. Devesa

**Duration**: February 2011 to January 2014

**Program**: Technical Personnel to Assist Research

**Funding body**: Ministry of Education and Vocational Training

**Reference**: PTA2017-13723-I

**Lead researcher**: J. A. Devesa

**Duration**: 2019−2021

Contracts from the Youth Employment Plan:

**Program**: National Youth Guarantee System

**Funding body**: Ministry of Employment and Social Security

**Lead researcher**: PEJ-2014-A-82677

**Researcher**: J. A. Devesa

**Duration**: 2016−2017

**Program**: National youth guarantee system and the youth employment operational program

**Funding body**: Junta de Andalucía

**Reference**: EJ-17-Herb

**Lead researcher**: J. A. Devesa

**Duration**: June 2017 to May 2018

**Program**: National youth guarantee system and the youth employment operational program

**Funding body**: Junta de Andalucía

**Reference**: EJI-17-RNM-260

**Lead researcher**: J. A. Devesa

**Duration**: January 2018 to July 2019

**Program**: National youth guarantee system and the youth employment operational program

**Funding body**: Ministry of Economy, Industry, and Competitivity, and Junta de Andalucía

**Reference**: EJI-17-RNM-260

**Lead researcher**: J. A. Devesa

**Duration**: March 2019 to February 2020

### Pending or delayed tasks

The following activities are being prioritized by the technical staff.

a) **Mounting, databasing, and inclusion of specimens from old collections**: About 10,000 sheets of unmounted and unfiled specimens that accumulated in the period 1977−1987 are pending allocation of a sheet number or accession number, as well as databasing and inclusion in the General collection. Also, some 4,000 sheets have been allocated an accession number, but their data have not been registered in the database, probably due to a lack of technical personnel during that period and/or the absence of standardized databases. All this material has been identified, and most of it has been mounted. It has been arranged by family in distinctive blue boxes; currently, it is being databased using Elysia v1.0.

These data were not available and so these specimens were not included in the taxonomic analysis or geographical coverage of this paper.

b) **Update the software and publication of data in GBIF**: First, the migration of the database to the new version 2.0 of the Elysia software (Pando et al. 2019) will be carried out. Regarding the publication of the data in GBIF, the records of *Festuca* need to be uploaded. This will be done once the study of its taxonomy and nomenclature has been completed (estimated date: December 2019). However, this paper has taken into account these records (811 so far) for the description of the collection.

## Interest and use of the collection

The General collection of COFC is fundamental for our knowledge of the Andalusian flora, and had great importance in the writing of the "Flora Vascular de Andalucía Occidental" (Valdés et al. 1987). It contains a vast representation of specimens from the province of Cordoba, whose study has deepened our knowledge of its flora, reflected in numerous publications in the last 20 years (e.g., Ruiz de Clavijo and Devesa 2010, López Tirado and Hidalgo 2014, López Tirado et al. 2013, 2015, Martínez-Sagarra et al. 2016, Muñoz et al. 2017, Reyes et al. 2017, Devesa et al. 2018, in press, among the most recent). It has also been fundamental for the taxonomic studies that have been carried out in the "Flora iberica" project (Castroviejo 1986−2019), especially from 2007 to the present, a period in which four subprojects have been developed, coordinated by Dr. J.A. Devesa from the University of Cordoba (projects CGL2008-02982-C03-03; CGL2011-28613-C03-02; CGL2014-52787-C3-3-P; CGL2017-85204-C3-3-P, of the Government of Spain). These focused mainly on the preparation of the taxonomic syntheses of the genera *Centaurea* (Devesa et al. 2014) and *Festuca* (taxonomic revision in progress), two of the most complex genera of the peninsular flora with an excellent representation in the collection. It is estimated that 16% of all the material preserved in the collection corresponds to duplicates, and these are especially common for the genera *Centaurea* (1,904 duplicates, Fig. 3B) and *Festuca* (1,906 duplicates, Fig. 3B), and for whose taxonomic synthesis abundant material was necessary in order to cover all their diversity, even at the population level (e.g., Invernón and Devesa 2013, Devesa 2016, Martínez-Sagarra and Devesa 2015, 2019, López et al. 2016, 2017). Besides, much of the material conserved in COFC also been used in morphological and biogeographic studies (Aedo et al. 2015, Cano et al. 2017, Devesa et al. in press), molecular and phylogeographic studies (Arnelas et al. 2018, Benítez-Benítez et al. 2018), foliar anatomy studies (Martínez-Sagarra et al. 2017), and genome size analysis (Martínez-Sagarra et al. work in progress).

In addition, 34 types (7 holotypes and 27 isotypes) of various described taxa of the Iberian Peninsula are preserved in COFC (Table 2).

**Table 2.** Type material of vascular plants conserved in COFC. Acronyms of the herbaria according to Thiers (2019). Holotypes and isotypes are listed in order of publication of the taxa.

Type	Taxon and authority	Publication	Herbarium voucher	Collection location (direct quote)
Holotype	*Centaurea susannae* Invernón & Devesa	Phytotaxa 74: 42, 43 (2012)	COFC 57935 (1 isotype in COFC)	Algarve, Cabo de San Vicente, 6 May 2010
Holotype	*Centaurea molesworthiae* E. López, Devesa & García Rojas	Nordic J. Bot. 30: 422 (2012)	COFC 59475 (1 isotype in COFC)	Cádiz, Tarifa, Sierra de Ojén, Loma de El Chivato, 19 June 2010
Holotype	Centaurea susannae var. paivae Invernón & Devesa	Acta Bot. Malac. 38: 66 (2013)	COFC 60956 (1 isotype in COFC)	Algarve, Aljezur, Monte Clérigo […], 5 June 2012
Holotype	*Centaurea stuessyi* Arnelas, Devesa & E. López	Phytotaxa 115: 43, 45 (2013)	COFC 57443 (1 isotype in COFC)	Tarragona, Rasquera, balneario de Cardó, 1 July 2009
Holotype	*Mantisalca cabezudoi* E. Ruiz & Devesa	Nordic J. Bot. 32: 18, 19 (2014)	COFC 31890 (1 isotype in COFC)	Granada, Jerez del Marquesado, barranco de Alcázar, 13 June 2007
Holotype	*Festuca greuteri* Martínez-Sagarra & Devesa	Phytotaxa 395: 253 (2019)	COFC 65825 (1 isotype in COFC)	Granada, Sierra de Guillimona, puerto de la Losa, 4 June 2014
Holotype	Festuca greuteri subsp. camarolensis Martínez-Sagarra & Devesa	Phytotaxa 395: 257 (2019)	COFC 65824 (9 isotypes in COFC)	Málaga, Villanueva del Rosario, Sierra de Camarolos, 10 June 2015
Isotype	*Erica andevalensis* Cabezudo & Rivera	Lagascalia 9: 224 (1980)	COFC 12422 (Holotype in SEV)	Huelva, Zalamea la Real, 21 July 1979
Isotype	Silybum × gonzaloi Cantó, Sánchez Mata & Rivas Mart. [*S. eburneum* Cosson & Durieu x *S. marianum* (L.) Gaertner]	Itin. Geobot. 15: 707 (2002)	COFC 61148 (Holotype in MAF)	Ciudad Real, Pedro Muñoz, laguna del Retamar, 8 May 2001
Isotype	Centaurea catellanoides subsp. talaverae E. López & Devesa	Acta Bot. Malac. 33: 60 (2008)	COFC 30665 (Holotype in UNEX)	Toledo, Noblejas, carretera en dirección a Dos Barrios, 7 July 2005
Isotype	*Centaurea beturica* E. López & Devesa	Anales Jard. Bot. Madrid 65: 334 (2008)	COFC 30719 (Holotype in UNEX)	Badajoz, camino hacia La Garganta, 10 July 2005
Isotype	Centaurea castellanoides subsp. arundana E. López & Devesa	Acta Bot. Malac. 3: 60 (2008)	COFC 30681. (Holotype in UNEX)	Málaga, Parque Natural de la Sierra de las Nieves […], 10 July 2004
Isotype	Centaurea langei subsp. dominguezii E. López, Devesa & Arnelas	Ann. Bot. Fenn. 48: 7 (2011)	COFC 30666 (Holotype in UNEX)	Salamanca, presa de Aldedávila de la Ribera, 1 July 2003
Isotype	*Orobanche subbaetica* Triano & A. Pujadas	Acta Bot. Malac. 39: 275 (2014)	COFC 61244 (Holotype in COA)	Córdoba, Las Angosturas, 22 May 2010
Isotype	*Foeniculum sanguineum* Triano & A. Pujadas	Acta Bot. Mal. 40: 75 (2015)	COFC 62161 (Holotype in COA)	Cádiz, Benamahoma, Sierra de Grazalema, 19 June 2006
Isotype	× *Tritordeum martinii* A. Pujadas [*Triticum durum* Desf. × *Hordeum chilense* Roem. & Schult.]	Acta Bot. Malac. 41: 327 (2016)	COFC 63411 (Holotype in COA)	Córdoba, La Carolota, finca El Patronato, 29 May 2016
Isotype	*Poa flaccidula subsp. guadianensis* F.M. Vázquez	Folia Bot. Extremad. 9: 66 (2016)	COFC 62937 (Holotype in HSS)	Badajoz, Nogales, Los Madroñales, 9 March 2015
Isotype	*Festuca discreta* F.M.Vázquez	Folia Bot. Extremad. 10: 66 (2016)	COFC 62911 (Holotype in HSS)	Badajoz, Puebla del Maestre, proximidades del rio Viar, 8 May 1998
Isotype	Narcissus nevadensis subsp. herrerae Algarra, Blanca, Cueto & J. Fuentes	Phytotaxa 371(2): 134 (2018)	COFC 66040 (Holotype in GDA)	Granada, Jayena, Sierra de Almijara, barranco de la Culebra, 9 April 2016

## Dataset description

**Object name**: Darwin Core Archive Dpto de Botánica, Ecología y Fisiología Vegetal (COFC). Facultad de Ciencias. Universidad de Córdoba

**Character encoding**: UTF-8

**Format name**: Darwin Core Archive format

**Format version**: 1.0

**Distribution**: https://doi.org/10.15468/fdzzal

**Publication date of data**: 2019-07-19

**Language**: Spanish

**Licences of use**: Creative Commons Attribution Non Commercial (CC-BY-NC) 4.0 License

**Metadata language**: Spanish

**Date of metadata creation**: 2019-06-18

**Hierarchy level**: Dataset
